# Zika Virus Transmission Through Blood Tissue Barriers

**DOI:** 10.3389/fmicb.2019.01465

**Published:** 2019-07-04

**Authors:** Svetlana F. Khaiboullina, Fabiola M. Ribeiro, Timsy Uppal, Ekaterina V. Martynova, Albert A. Rizvanov, Subhash C. Verma

**Affiliations:** ^1^Department of Microbiology and Immunology, Reno School of Medicine, University of Nevada, Reno, Reno, NV, United States; ^2^Department of Exploratory Research, Scientific and Educational Center of Pharmaceutics, Kazan Federal University, Kazan, Russia; ^3^Department of Biochemistry and Immunology, Universidade Federal de Minas Gerais, Belo Horizonte, Brazil

**Keywords:** ZIKV, ZIKV transmission, blood tissue barriers, placenta, microcephaly

## Abstract

The recent Zika virus (ZIKV) epidemic in the Americas and the Caribbean revealed a new deadly strain of the mosquito-borne virus, which has never been associated with previous outbreaks in Asia. For the first time, widespread ZIKV infection was shown to cause microcephaly and death of newborns, which was most likely due to the mutation acquired during the large outbreak recorded in French Polynesia in 2013–2014. Productive ZIKV replication and persistence has been demonstrated in placenta and fetal brains. Possible association between ZIKV and microcephaly and fetal death has been confirmed using immunocompetent mouse models *in vitro* and *in vivo*. Having crossed the placenta, ZIKV directly targets neural progenitor cells (NPCs) in developing human fetus and triggers apoptosis. The embryonic endothelial cells are exceptionally susceptible to ZIKV infection, which causes cell death and tissue necrosis. On the contrary, ZIKV infection does not affect the adult brain microvascular cell morphology and blood–brain barrier function. ZIKV is transmitted primarily by *Aedes* mosquito bite and is introduced into the placenta/blood through replication at the site of the entry. Also, virus can be transmitted through unprotected sex. Although, multiple possible routes of virus infection have been identified, the exact mechanism(s) utilized by ZIKV to cross the placenta still remain largely unknown. In this review, the current understanding of ZIKV infection and transmission through the placental and brain barriers is summarized.

## Epidemiology

For decades, Zika virus (ZIKV) sporadic outbreaks have been registered in several countries in Asia, and the infection has been characterized by mild symptoms, where up to 80% of cases remain asymptomatic (Duffy et al., 2009). However, the 2014 outbreak in the South/Central Americas and the Caribbean opened a new chapter in ZIKV epidemiology, where neurological complications were demonstrated in adults and newborns (Triunfol, 2016; Roze et al., 2017; Lowe et al., 2018). The most alarming news was that nearly 10% of ZIKV infection cases were reported to cause microcephaly or fetal death (CDC, 2017) with devastating impact on families and communities of infected individuals (de Oliveira et al., 2017). Targeting the fetal brain tissues and death of neural progenitor cells (NPCs) by ZIKV has been confirmed in several research studies using immunocompetent animal models (Dong and Liang, 2018). ZIKV neurotropism seems to be quite puzzling since neuropathology has never been a characteristic associated with earlier outbreaks in South East Asia and Africa (Lim et al., 2017). It now appears that a single mutation (serine to asparagine, S139N) in the viral precursor membrane protein (prM) made the relatively mild virus to a more pathogenic and deadlier strain (Yuan et al., 2017). It has been suggested that this mutation arose during the 2013 ZIKV outbreak in Polynesia (Yuan et al., 2017). The association between microcephaly and prM mutation was further confirmed in a retrospective study, where clusters of microcephaly cases were identified in French Polynesia during the ZIKV outbreak (Cauchemez et al., 2016; Baud et al., 2017). In addition to the mutation in ZIKV genome, socioeconomic condition was also suggested to contribute to the development of microcephaly (Franca et al., 2018; Souza et al., 2018). Analysis of the spatial distribution revealed that the majority of microcephaly was diagnosed in economically deprived areas, while only 2% of cases were located in the wealthiest district (Souza et al., 2018). Therefore, it could be suggested that mutation in ZIKV genome combined with other factors could contribute to the development of microcephaly.

The first ZIKV strain was isolated from non-human primates in 1947 from sylvatic mosquitoes in 1948 (Dick, 1952). Since, only a few human cases were reported after ZIKV discovery, virus was not considered a public health threat (Macnamara, 1954) until 2007, when the first ZIKV epidemic was reported in Micronesia (Duffy et al., 2009) where nearly 73% of the inhabitants were reported to be infected. This outbreak was followed by another large epidemic in French Polynesia (2013–2014, Cao-Lormeau et al., 2014). The most disturbing features of these outbreaks were microcephaly and Guillain-Barre Syndrome (GBS) linked to ZIKV infection (Cao-Lormeau et al., 2016; Cauchemez et al., 2016; Johansson et al., 2016). Cao-Lormeau et al. (2016) suggested that the pathogenesis of GBS is complex, including HLA allele genotype, autoantibodies and unknown neurotoxic factors. Later, GBS cases were reported during the outbreak in several South American countries, supporting the link between ZIKV infection and the disease (European Centre for Disease Prevention, and Control, 2016). GBS is an inflammatory neurological disease, which is associated with the post-infection nerve injury (Wijdicks and Klein, 2017). The pathogenesis of GBS remains largely unknown; however, it appears that ZIKV infection often triggers the onset of the disease (Wijdicks and Klein, 2017).

In a short duration, ZIKV crossed the Pacific Ocean, reaching the South America in 2014 (Lowe et al., 2018). An outbreak in Brazil was followed by reports of several ZIKV infection cases in numerous South American countries (Heukelbach et al., 2016; Pan American Health Organization World Health Organization, 2016). By the year 2017, approximately 217,000 confirmed ZIKV cases were reported to the Pan-American Health Organization (PAHO, 2016). It appears that ZIKV epidemic reached its peak in 2016, when a large number of suspected/confirmed ZIKV cases were reported (PAHO, 2017). However, in 2016, ZIKV incidence declined in all South American countries (PAHO, 2017). A similar pattern of ZIKV epidemic has been observed in the United States, where the highest number of ZIKV cases (5,168 symptomatic diseases) were documented in the year 2016 (CDC, 2016). However, ZIKV infection reports declined in 2017, when only 452 cases were documented. Even fewer ZIKV infections were confirmed by August of 2018 (34 cases).

Like many *Flaviviruses*, ZIKV is transmitted by infected *Aedes* species mosquitoes (*Aedes aegypti* and *Aedes albopictus*), which are present across the large territories including Africa, South/South-East Asia, and South/North America. The distinct feature of *A. aegypti* is that they feed almost exclusively on humans and usually rest indoors (Scott and Takken, 2012; Kraemer et al., 2015). It is believed that factors, such as human activities and climate changes, contribute to geographical expansion of mosquitoes/virus currently spread-out in Europe, United States, and South America (Medlock et al., 2012; Carvalho et al., 2014).

Zika virus emerges in regions also known to be endemic for Dengue virus (DENV) (Rigau-Pérez et al., 1998). Also, ZIKV was shown to have structural homology with DENV (Sirohi et al., 2016; Prasad et al., 2017), which could explain high antibody cross reactivity (Lanciotti et al., 2008; Duffy et al., 2009; Stettler et al., 2016). Antibody cross reactivity plays role in DENV pathogenesis and is linked to dengue hemorrhagic fever and fatality (Guzman et al., 1987). Therefore, it was suggested that *flaviviridae* antibody cross reactivity could play role in pathogenesis of ZIKV. However, data on the role of the cross-reactive antibodies in ZIKV pathogenesis is controversial. Brown et al. (2019) have demonstrated that this cross reactivity plays a role in enhancing the severity of ZIKV infection. Using an animal model, Brown et al. (2019) have demonstrated that DENV antibodies could increase placental damage, fetal growth restriction, and fetal resorption. These data suggest the detrimental role of antibody cross reactivity in the recent ZIKV outbreak in South America. In contrast, studies have shown that flaviviruses could induce a cross protective immune response. For example, Grifoni et al. (2017) have demonstrated that previous DENV infection could elicit memory T cells recognizing ZIKV peptides. Authors suggested that prior DENV exposure could protect against ZIKV infection by affecting the timing, magnitude, and quality of the T cell response. Additionally, Ribeiro et al. (2018) suggested that ZIKV cross-reacting antibodies may protect against DENV, as a significantly decreased frequency of DENV infection was documented after ZIKV outbreak in South America. Therefore, co-circulation of two flaviviruses sharing multiple immunogenic epitopes could affect the epidemiology of each virus; although the outcomes of the immunological cross reactivity in ZIKV and DENV remain to be determined.

## Genomic Structure

The first ZIKV strain was isolated in 1947 from a sentinel monkey in Zika forest in Uganda, Africa (Dick et al., 1952). Later, many new strains were identified which are divided into two major lineages: African and Asian (including South American) (Haddow et al., 2012; Gong et al., 2016). The origin of these two lineages is still debated. It was suggested that both ZIKV lineages originated from the strain isolated in Uganda in 1947 (Yokoyama and Starmer, 2017). However, Gong et al. (2016) proposed that the African strains diverged to produce distinct African and Asian lineages.

**FIGURE 1:**
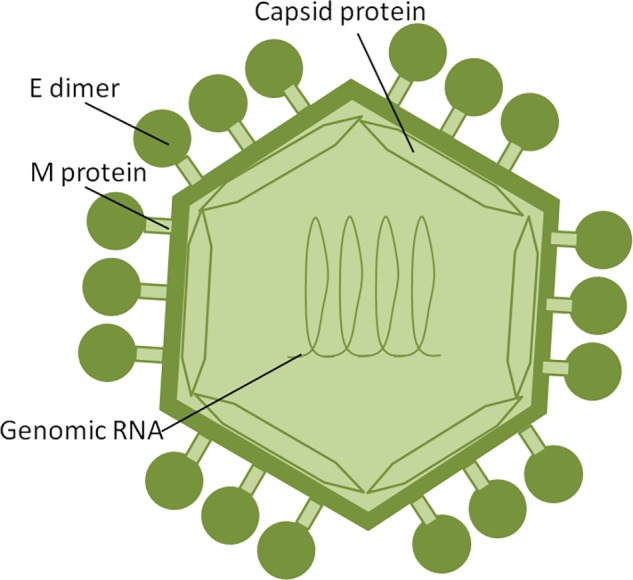
ZIKV structure. ZIKV RNA is enclosed within thecapsid shell and envelope. Envelope protein dimers are located on the virion surface. The M protein is hidden under theenvelope proteins.

**FIGURE 2:**
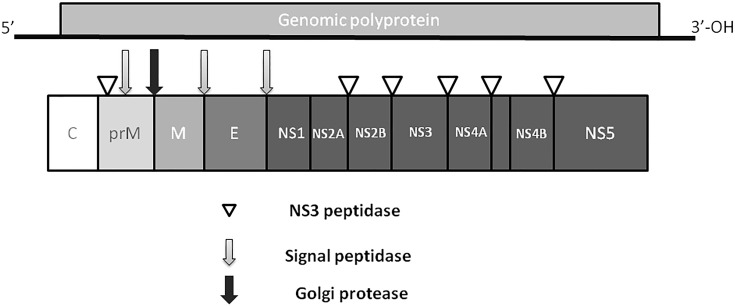
ZIKV genome. Virus genome consists of monopartite, linear single stranded, positive sense RNA. RNA is translated into single polyprotein, which is post-translationally cleaved releasing three structural proteins, capsid (C), prM,envelope (E), and five non-structural proteins (NS1, NS2, NS3, NS4 and NS5). prM protein is cleaved by Golgi peptidase,releasing mature M protein and prM. Later, NS2 and NS4 are cleaved by NS3 to release NS2A, NS2B, NS4A and NS4B proteins.

Zika virus is a member of the *Flavivirus* family, which also includes several human pathogens such as West Nile Virus (WNV), DENV and Yellow Fever Virus (YFV). Kostyuchenko et al. (2016) have shown that structurally ZIKV is similar to DENV and WNV. However, ZIKV is a thermally stable virus compared to DENV and its stability at 40°C is considered a key factor behind the epidemics in the temperate zones (i.e., Brazil/South America). ZIKV genome is composed of a positive single-stranded RNA, which is protected by the capsid and an envelope layers (Hasan et al., 2018) (Figure 1). The viral RNA is translated into a single polypeptide, which is cleaved into three structural proteins, (premembrane/membrane-prM/M, Envelope/E and Capsid/C) and seven non-structural (NS) proteins (NS1, NS2A, NS2B, NS3, NS4A, NS4B, and NS5) by viral and host proteases (Figure 2). The E protein binds to the receptor and fuses with the cell membrane. Sequence and structural analysis of ZIKV E protein revealed that some parts of the protein have a close resemblance to neurovirulent WNV and Japanese encephalitis viruses (JEV), while others are similar to DENV (Kostyuchenko et al., 2016). This may explain the neurological symptoms in ZIKV- infected patients as well as cross-reactivity between the members of *Flaviviridae* family. The M protein is hidden under the E protein/layer. The mature M protein is released by cleavage of prM protein by furin in the Golgi (Schlesinger et al., 1989). The prM acts as a chaperone for the E protein during virion assembly (Roby et al., 2015). It was shown that a single mutation in the prM protein converted a relatively mild ZIKV strain into a teratogenic strain (Yuan et al., 2017).

Five NS proteins are encoded by viral RNA, where two of them (NS1 and NS4) are post- translationally cleaved making a total of seven NS proteins. Each NS protein plays a unique role in virus replication and evasion of the host immune responses. NS1, a strong immunogen (Muller and Young, 2013), co-localizes with viral dsRNA and plays an important role in virus replication (Muller and Young, 2013) and interferon-associated antiviral defense (Xia et al., 2018). The NS2 and NS3 proteins are shown to play a crucial role during virion assembly (Leung et al., 2008; Sahoo et al., 2016). In addition, NS3 protein, together with NS2B protein, functions as a protease, RNA helicase, nucleotide triphosphatase, and RNA triphosphatase (Wengler et al., 1991; Warrener et al., 1993; Li et al., 1999; Yusof et al., 2000). The NS4 protein interferes with intracellular signaling and has been linked to autophagy and altered neurogenesis (Liang et al., 2016). The NS5 protein was shown to act as a methyltransferase, guanylyltransferase, and RNA-dependent RNA polymerase (Egloff et al., 2002; Ray et al., 2006; Dong et al., 2012).

## Modes of Transmission

### Cell Tropism

Clinical presentations of ZIKV infection vary from mild skin rashes to severe brain damage and auto-immune disorder (Calvet et al., 2016), which could be attributed to the fact that ZIKV can target multiple cell types. Two receptors, AXL and TIM1, were suggested as candidates for ZIKV entry *in vitro*, while physiological relevance of these receptors is yet to be determined *in vivo* (Retallack et al., 2016; Tabata et al., 2016). Expression of AXL, a member of the TAM receptor family of cell surface receptor tyrosine kinases (Wu et al., 2014), has been linked to ZIKV entry in multiple cell types (Meertens et al., 2017). This receptor is expressed on various cells including keratinocytes, endothelial cells, and NPCs (Hamel et al., 2015; Nowakowski et al., 2016; Richard et al., 2017). It appears that the tyrosine kinase activity of AXL is important for viral entry *in vitro* (Liu et al., 2016). Interestingly, Richard et al. (2017) have shown that AXL-dependent infection of fetal endothelial cells is a unique feature of ZIKV infection that could explain virus tropism to fetus tissues. However, it appears that although AXL receptor is essential for ZIKV entry *in vitro*, its role in *in vivo* remains controversial. Hastings et al. (2017) demonstrated that susceptibility of ZIKV infection in *Axl*^-/-^ mice remains similar to that of wild type animals. Similarly, Wang et al. (2017) revealed that there is no difference in the survival rate, clinical manifestations, viral load and ZIKV distribution among *Axl*- mice and wild type control animals. Collectively, data suggest that AXL receptors are indispensable for ZIKV entry and other receptors may be involved as well.

Several cell types were shown to be susceptible to ZIKV infection, including endothelial cells, monocytes, skin keratinocytes, dendritic cells and NPCs (Hamel et al., 2015; Bowen et al., 2017; Devhare et al., 2017; Khaiboullina et al., 2017; Richard et al., 2017). Two independent comprehensive studies have demonstrated susceptibility of multiple cell types derived from lung, liver, skin, kidney, ovary, retina, prostate, muscle tissue, nervous tissue and testicular tissue to ZIKV infection (Chan et al., 2016; Himmelsbach and Hildt, 2018). While all of these cell types were found to be susceptible to ZIKV infection, virus replication was found to be the lowest in nervous tissue derived cells. Also, Chan et al. (2016) have shown that ZIKV replication is cytopathic in neuronal cells, which could explain the neurological damage in the fetus. In addition, cytopathic effect has been demonstrated in the placental, but not in prostatic, testicular and renal cell lines. Authors suggested that the cytopathic effect of ZIKV in placental cells could explain the teratogenic effect of the virus. Also, the lack of cytopathic effect, combined with the effective virus replication in prostatic and testicular cells, were suggested as key reasons for the virus shedding and sexual transmission in ZIKV-infected individuals.

Zika virus crosses placenta, reaching the fetus to target the neuronal tissue (Adibi et al., 2016). Therefore, identification of cells that support ZIKV replication in the placenta is especially important to understand the mechanisms utilized by the virus to cross the placenta and to further design anti-ZIKV drugs. Several cell types within the umbilical cord and placenta were shown to be susceptible to ZIKV infection (El Costa et al., 2016). Among the cell types studied, umbilical mesenchymal cells effectively supported virus replication (El Costa et al., 2016). Interestingly, these cells were able to facilitate the virus spread *via* cell-to-cell contact, suggesting that binding to the receptor may not be the only way that virus spreads within the tissue. Additionally, decidual macrophages from placenta are also susceptible to ZIKV infection, supporting the data published by Quicke et al. (2016) demonstrating that placental macrophages are key players in ZIKV transmission.

Multiple studies have shown that endothelial cells support ZIKV infection and replication (Quicke et al., 2016; Papa et al., 2017; Roach and Alcendor, 2017; Alimonti et al., 2018; Peng et al., 2018). Endothelial cells are an essential component of the blood–tissue barrier controlling the leukocyte trafficking and solute permeability. It appears that ZIKV has limited effect on brain endothelial monolayer permeability *in vivo* (Papa et al., 2017). This data provides an explanation of the limited effect of ZIKV infection in the adult’s brain, while mechanisms of virus spread to the fetal brain still remain elusive.

Zika virus can infect various populations of leukocytes. We have shown that ZIKV infects human monocytes (Khaiboullina et al., 2017). It appears that monocytes are the main leukocyte population harboring the virus in circulation (Michlmayr et al., 2017). Monocytes are a mobile population of leukocytes capable of traveling across the endothelium.

Monocytes trans-endothelial migration could contribute to the virus dissemination across the placenta, as has been shown for other viruses targeting the fetus (Arias et al., 2003; Pereira et al., 2003). Also, ZIKV replication causes activation of the inflammasome (Khaiboullina et al., 2017), a complex structure that is assembled upon the detection of various danger signals (Horvath et al., 2011). IL-1β is a gene product of the activated inflammasome (Martinon et al., 2002; Wang et al., 2002), which is a proinflammatory cytokine regulating lymphocyte differentiation (Garlanda et al., 2013). This cytokine appears to be indispensable for protection against several infectious agents (Sansonetti et al., 2000; Vonk et al., 2006; Miller et al., 2007). However, the excessive production of IL-1β could cause devastating consequences, damaging tissues and triggering autoimmunity (Wan et al., 2016). Increased IL-1β in ZIKV infected cells could be further investigated to determine its exact role in tissue damage and GBS pathogenesis.

### Tissue–Brain Barrier Permeability

The integrity of the endothelial monolayer is essential for the functional capacity of the blood–tissue barrier. The barrier function is based on selective permeability, where the crossing of molecules and cells is restricted (Rodrigues and Granger, 2015). Endothelial cells act as a barrier for microbial pathogens by triggering inflammatory and coagulatory responses upon antigen recognition (Valbuena and Walker, 2006). Therefore, crossing the endothelial barrier is a crucial step for microbial dissemination and reaching the target organ. Endothelial cell death is most commonly identified as the cause of blood–tissue barrier insufficiency during viral infection (Lunardi et al., 2007; Long et al., 2013; Lin et al., 2014). Viruses target endothelium, which serves as the initial site of virus propagation before disseminating across the blood–tissue barrier. Flaviviruses infect and propagate in endothelial cells (Khaiboullina et al., 2005; Dalrymple and Mackow, 2011), including ZIKV (Mladinich et al., 2017; Roach and Alcendor, 2017; Peng et al., 2018). It has been suggested that ZIKV replication could affect endothelial integrity, compromising the blood–tissue barriers (Garcez et al., 2018). Therefore, the susceptibility of brain endothelial cells to ZIKV is particularly important, since virus targets the fetus neural tissue (Chan et al., 2016). By crossing fetal blood–brain barriers (BBB) and affecting blood vessel development, ZIKV could interfere with brain development and cause tissue damage. A report supporting this assumption was published by Shao et al. (2016), where abnormal density and diameter of the brain blood vessels as well as a leaky BBB were demonstrated in ZIKV infected fetus. It appears that ZIKV infection could cause embryonic endothelial cell necrosis (Szaba et al., 2018), which could help explain the fetal brain damage. Interestingly, the effect of ZIKV on endothelial cell permeability *in vivo* contrasts with data collected using an *in vitro* model (Mladinich et al., 2017). It appears that ZIKV infection does not affect brain endothelial cell monolayer permeability *in vitro*, although it can actively replicate and produce infectious virus (Mladinich et al., 2017). It could be suggested that the *in vitro* model represents an isolated system, which is limited to infection of the endothelial cells. In contrast, *in vivo* models include interaction between ZIKV infected endothelial cells and surrounding tissue as well as the leukocytes.

### *Aedes* Mosquito Bite Transmission

**FIGURE 3:**
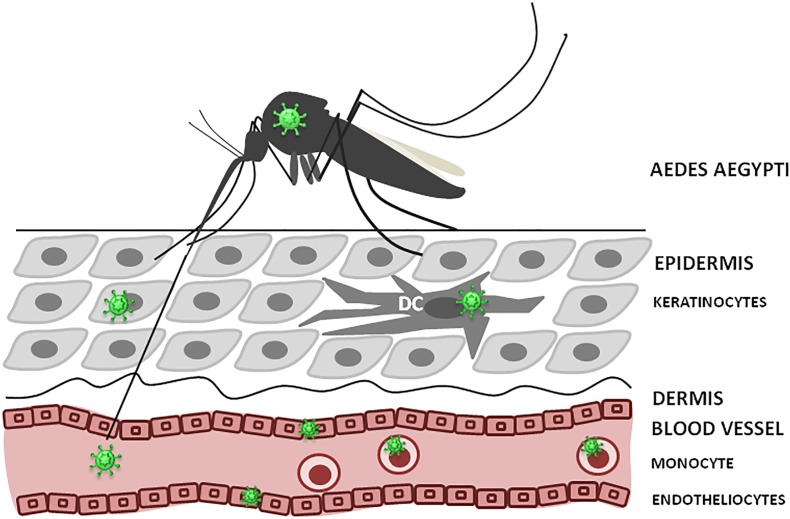
ZIKV infection, initial replication and dissemination. ZIKV is transmitted by *Aedes* mosquito into theepidermis or directly into the blood. Keratinocytes, dendritic cells (DC) and endothelial cells could support virus replication at the entry site. Also, circulating monocytes could become infected and facilitate virus dissemination.

As, ZIKV is primarily transmitted to people through the bites of infected female *Aedes* species mosquitoes, hence, understanding ZIKV pathogenesis relies on identification of the cellular targets of ZIKV infection, which is essential to trace virus dissemination outside of the uterus (Figure 3). Similar to other flaviviruses, the dendritic cells and endothelial cells appear to be the initial targets for ZIKV. Keratinocytes also serve as the initial site for virus replication, from which the virus spreads to endothelial cells where further virus propagation occurs. From the site of initial replication, virus disseminates *via* blood vessels, reaching the uterus, testicles, brain and other organs during viremia. Interestingly, ZIKV can target many immune privileged organs, such as brain, placenta and testicles, for which virus should cross blood–tissue barriers that are designed to restrict the tissue access for leukocytes and solutes. Endothelial cells are the essential structural and functional components of the BBB. Therefore, it could be suggested that by propagation in these cells, virus makes the first and the most crucial step in crossing into the tissue. Another potential mechanism for virus dissemination and crossing of the tissue barrier could be *via* circulating leukocytes acting as the carrier. This assumption is supported by our finding of monocytes harboring ZIKV (Khaiboullina et al., 2017). Also, monocyte susceptibility to ZIKV infection was demonstrated by Foo et al. (2017). These leukocytes were shown to be the main population carrying ZIKV in circulation (Michlmayr et al., 2017). However, many intricate details of virus trans-endothelial migration still remain unknown.

### Sexual Transmission

In addition to the well characterized mosquito-borne ZIKV transmission, there are several case reports indicating that the virus can also be transmitted through sexual contact. The first case of ZIKV sexual transmission was reported in 2008 in Colorado, United States. A female patient who had not traveled to endemic areas exhibited typical symptoms of ZIKV infection. Patient reported that she had unprotected sexual intercourse with her husband, who had just returned from Senegal and was diagnosed positive for ZIKV (Foy et al., 2011). Since then, several reports indicated that ZIKV could be transmitted sexually. Most of these studies report ZIKV transmission from male-to-female partners (D’Ortenzio et al., 2016; Hills et al., 2016; McCarthy, 2016; Venturi et al., 2016), although female-to-male (Davidson et al., 2016) and male-to-male (Deckard et al., 2016) sexual transmission of ZIKV have also been reported. Notably, it has been estimated in a systematic review that male to female (92.5%) sexual transmission of ZIKV is much more common than that of male to male (3.7%) and female to male (3.7%) (Moreira et al., 2017). Moreover, this study also points to the main mode of sexual transmission being the unprotected vaginal intercourse (96.2%), followed by oral (18.5%) and anal (7.4%) contacts (Moreira et al., 2017). In these reported cases, sexual transmission was considered as the most likely source of infection, since ZIKV infected patients lived in *Aedes* mosquitoes-free regions and had unprotected sex with the partners returning from ZIKV infected areas. In most of these reported cases, transmission occurred from a patient exhibiting symptoms of ZIKV infection. However, two cases, possibly of male-to-female sexual transmission of ZIKV involving asymptomatic men, have also been reported (Brooks et al., 2016; Freour et al., 2016). These findings have very profound implications in the dynamics of ZIKV transmission, especially concerning pregnant women or those considering pregnancy. Moreover, sexual transmission could be an important factor contributing to the spread of ZIKV into new geographical areas. For instance, the Centers for Disease Control and Prevention (CDC) reported 45 cases of sexually acquired ZIKV in 2016 and seven cases in 2017 in the United States [CDC 2018 Zika Virus: 2017 Case Counts in the United States (accessed on 9 May 2018, CDC, 2018)].

Since ZIKV is present in the semen at higher levels than in urine and blood, detected by the viral RNA (Atkinson et al., 2016; Barzon et al., 2016b; D’Ortenzio et al., 2016; Mansuy et al., 2016a,b; Matheron et al., 2016; Nicastri et al., 2016), further indicated sexual transmission being the relevant route of virus transmission. It is yet not clear why ZIKV is more concentrated in semen, but it was hypothesized that virus could replicate more efficiently in the male reproductive tract (Lazear et al., 2016). The time duration for viral persistence in semen is still a matter of debate. ZIKV sexual transmission has been reported to occur 41 days following disease onset (Turmel et al., 2016). Moreover, semen of a patient returning from Haiti was positive for ZIKV RNA even 6 months after the onset of symptoms (Barzon et al., 2016b; Nicastri et al., 2016). However, infectious ZIKV could only be detected in semen up to 69 days post-infection (Arsuaga et al., 2016). In addition, a cohort study analyzing 150 patients indicates that only a small percentage of male patients showed detectable ZIKV RNA 3 months of post-infection (Paz-Bailey et al., 2017). Moreover, ZIKV RNA was detected in semen from 30 to 50% of infected men in the first month after disease onset (Musso et al., 2017a; Paz-Bailey et al., 2017). These data together suggest that sexual transmission is more likely to occur early after infection.

Regarding the female reproductive tract, ZIKV was detected in genital swab specimens from a woman’s cervical mucous collected 11 days after illness onset, although urine and blood were no longer positive at this time point (Prisant et al., 2016). However, a cohort study demonstrated that only 1 out of 50 ZIKV-infected women were positive for ZIKV RNA in vaginal secretions (Paz-Bailey et al., 2017). To further understand the mechanisms underlying ZIKV infection, animal models of ZIKV intra-vaginal and sexual transmission have been developed. For instance, ZIKV was detected in rhesus macaque vaginal fluid of three non-pregnant females up to 7 days following subcutaneous inoculation (Dudley et al., 2016). Another study employing rhesus macaque indicates that both intravaginal and subcutaneous inoculation of ZIKV leads to persistent (up to 60 days post-infection) ZIKV infection in various anatomical locations, including heart, kidney, lymph nodes, and brain. Also ZIKV was detected in the vagina 8 days after intravaginal infection (Woollard et al., 2018). When female mice lacking type-I interferon (IFN) receptor were subcutaneously inoculated with ZIKV at embryonic day 6.5 or 7.5 (E6.5 or E7.5), the result was fetal demise associated with virus infection of the placenta and fetal brain (Miner et al., 2016). Moreover, wild type female mice subjected to intravaginal inoculation of ZIKV exhibited persistent viral RNA for 1–4 days post-infection, although RNA levels were no longer detected 7 days post-infection, and mice fully recovered (Yockey et al., 2016). However, type-I IFN receptor knockout mice challenged with ZIKV into the vagina demonstrated high levels of local ZIKV replication, systemic infection and death 9 days post-infection (Yockey et al., 2016). Another study, utilizing type-I IFN receptor knockout mice, has shown that males can transmit ZIKV to females through sexual contact at 3 days post-infection (Uraki et al., 2017). Moreover, it has been shown that interferon α/β and -γ receptor knockout AG129 mice, which are both type-I and II IFN signaling incompetent, can transmit ZIKV sexually from the male to the female (Duggal et al., 2017). In fact, ZIKV infection was observed in half of the AG129 female mice mated to infected AG129 males during days 7 through 19 after inoculation (Duggal et al., 2017). Sexual transmission has also been tested in a mouse model of ZIKV, i.e., the Rag1^-/-^ mice treated with αIFNAR1 antibody (AIR mice), that exhibits lower levels of viral proliferation (Winkler et al., 2017a). Although disease progression was slower in this mouse model, male-to-female sexual transmission was observed when type-I IFN receptor knockout females were mated with infected AIR males 8–12 days post-infection (Winkler et al., 2017b). Moreover, sexual transmission of ZIKV in this model led to infection of the uterus and placenta of mice that became pregnant, although fetuses were spared (Winkler et al., 2017b). It is still unclear what factors could protect females from ZIKV intravaginal infection. However, it was shown that progesterone levels appear to impact intravaginal infection, since administration of ZIKV into the vagina of AG129 mice triggers viral systemic infection and death when infection takes place in the diestrus-like phase, although estrus-like mice are resistant to ZIKV intra-vaginal infection (Tang et al., 2016). Moreover, administration of Depoprovera (medroxyprogesterone) to rhesus macaques allows preferential replication of ZIKV in the female reproductive tract following intravaginal transmission, but not after subcutaneous transmission (Carroll et al., 2017).

### Secondary Modes of Transmission

Zika virus could be detected in several body fluids including blood, breast milk, saliva, urine, sweat and tears (Calvet et al., 2018). Therefore, a secondary mode of ZIKV transmission was suggested, in which virus could be passed during the blood transfusion or breast feeding.

#### Blood

Blood samples are reported to be consistently positive for ZIKV, although viremia is transient and virus titer is lower than in urine or sperm (Lanciotti et al., 2008). Up to 28.1% of patients were reported to have a viremia (Musso et al., 2017b), where 3% were shown clinically asymptomatic (Musso et al., 2017b). Since the majority of ZIKV infected cases are asymptomatic, the risk of infection via blood transfusion remains high. It became an important public safety concern when ZIKV transmission via blood transfusion was reported by Barjas-Castro et al. (2016). The alarming news of ZIKV infection via blood transfusion prompted the US Food and Drug administration to recommend blood donor and blood product screening to reduce the risk of infection (Center for Biologics Evaluation and Research, 2018).

#### Breast Milk

Breastfeeding was suggested as a mode of ZIKV infection in three clinical cases (Colt et al., 2017). In all three cases, ZIKV was detected in the breast milk by RT-PCR. ZIKV infection was confirmed in two breastfed newborns, where symptoms were limited to maculopapular rash without adverse effects on the brain growth or function. In a comprehensive systemic review, Colt et al. (2017) conclude that the data on breast milk transmission of ZIKV is insufficient and render more clinical data collection for the analysis. Although ZIKV transmission via breast milk was suggested in 2 cases, breastfeeding is still considered to be safe for the newborns. Therefore, WHO does not impose restrictions on the breastfeeding guidelines and recommends initiating breastfeeding within 1 hour of delivery (WHO, 2018).

#### Saliva

Saliva appears to be the excellent source for ZIKV detection where 57.1% samples were found positive for virus antigen (Musso et al., 2015). Interestingly, ZIKV detection in saliva was twice that in the blood (28.1%), suggesting that salivary glands are the sites of ZIKV replication. These data suggest that saliva could be the source of ZIKV transmission, especially in the endemic areas. Additionally, saliva transmission could play a role in sporadic outbreaks in non-endemic areas. Barzon et al. (2016a) reported detection of ZIKV in saliva of an Italian traveler returning from Dominican Republic. ZIKV was detected in saliva samples up to 29 days after the disease onset and it remained detectible longer than in blood. Therefore, ZIKV in saliva could contribute to the local epidemic as well as outbreaks in non-endemic areas.

#### Sweat and Tears

Zika virus can cause conjunctivitis and uveitis in up to 15% of infected adults (Furtado et al., 2016; Petersen et al., 2016), suggesting that eyes could be a site of virus replication. ZIKV was detected in conjunctival swabs from 10% of convalescent patients 30 days after the recovery. Although viral copies were low (5.2 to 9.3 copies per swab), detection of the virus in tear samples late after the recovery suggests that virus could establish long-term replication in selected sites. Interestingly, presumptive tears or sweat as a source of infection was suggested in a ZIKV case published by Swaminathan et al. (2016). Although this is the single study where tear/sweat source of infection was suggested, the data presents these sources as plausible and playing a role in the ZIKV epidemic.

#### Urine

Zika virus is consistently demonstrated in all urine samples studied by Gourinat et al. (2015). ZIKV nucleic acid was detected for up to 20 days after the onset of symptoms, which was longer than that in blood samples. Even longer, 21 days after the onset of neurological symptoms, virus shedding was demonstrated in patients diagnosed with GBS (Roze et al., 2016). The late detection of ZIKV RNA in the urine was reported by Campos Rde et al. (2016), suggesting that virus could be replicating in kidney tissue. This assumption is supported by the fact that ZIKV RNA could be found in urine long after the virus has being cleared from the circulation and no longer detected in the blood (Gourinat et al., 2015). ZIKV in the urine remains infectious, as several isolates from urine samples were established. The first ZIKV isolate was described in 2014 by Fonseca et al. (2014). Later, more ZIKV isolates were characterized (Bonaldo et al., 2016), including a travel-associated ZIKV, reported by Barzon et al. (2016a). Although infectious virus is readily detected in the urine, there is no documented case of urine exposure linked ZIKV infection. Nevertheless, urine samples should be considered as a potential source of infection and treated as biohazard material.

### Risk of Congenital Transmission Due to Intravaginal ZIKV Infection

Congenital ZIKV infection can cause microcephaly and severe brain abnormalities (de Araujo et al., 2016; Schuler-Faccini et al., 2016). The Congenital Zika Syndrome (CZS), i.e., neuropathological conditions that emerge after ZIKV intrauterine transmission, is characterized by severe microcephaly, a partially collapsed skull, decreased brain tissue with a specific pattern of brain damage and calcifications, eye alterations, congenital contractures, and hypertonia restricting body movements (Moore et al., 2017). However, not all pregnant women infected with ZIKV give birth to babies with CZS. For instance, among 117 babies born from symptomatic ZIKV-positive mothers in Rio de Janeiro, 49 (42%) presented with abnormal clinical and/or brain imaging findings, including 4 infants with microcephaly (Brasil et al., 2016). Another report by Reynolds et al. (2017) analyzed 972 fetuses/infants from completed pregnancies with laboratory evidence of possible ZIKV infection and identified 51 fetuses/infants (5%) exhibiting ZIKV-related birth defects. However, when only laboratory-confirmed ZIKV cases were studied, a higher proportion of fetuses/infants (24 out of 250, 10%) was found to have ZIKV-related birth defects (Reynolds et al., 2017). When ZIKV infection occurred in the first trimester of pregnancy, birth defects were reported in 15% of fetuses/infants (Reynolds et al., 2017). Moreover, another study estimated the risk of microcephaly due to ZIKV infection between 0.88 and 13.2% in cases of viral infection in the first trimester of pregnancy (Johansson et al., 2016). Compared to the prevalence during pre-ZIKV years, the proportion of fetuses/infants displaying birth defects among pregnancies with confirmed ZIKV infection was 30 times higher when infection took place at any time during pregnancy and even higher when infection occurred in the first trimester (Cragan et al., 2017). Moreover, it appears that birth defects occur regardless of whether infections during the pregnancy are symptomatic or asymptomatic (Cragan et al., 2017). Thus, these findings suggest that all pregnant women should be screened for possible ZIKV exposure to facilitate appropriate intervention.

As only a fraction of infected pregnant women give birth to babies with birth defects, it is possible that additional factors might contribute to ZIKV-triggered neurological alterations. Several factors have been suggested to play a role in ZIKV-related birth defects, including poor living conditions (Souza et al., 2018). For instance, from November 2015 to February 2016, out of the 1,501 livebirths registered with microcephaly, approximately 90% were born in the Northeast of Brazil, a region that faces many socio-economic problems and poor nutrition (Franca et al., 2016). In Recife (Pernambuco, Brazil), a city that was severely affected by ZIKV congenital malformations, the majority of the microcephaly cases in 2015 and 2016 were reported in areas with very impoverished living conditions (Nem de Oliveira Souza et al., 2018). However, there are many factors that complicate the interpretation of the correlation between poor living conditions and the prevalence of CZS. For example, deprived areas are overcrowded and lack basic sanitation, providing an ideal environment for transmission of vector-borne diseases (Braga et al., 2010). Thus, it is possible that CZS could be more common in these areas because of the high prevalence of ZIKV infection itself.

Although it is very hard to measure sexual transmission in endemic areas, epidemiological studies have reported increased incidence of ZIKV infections in pregnant women during the outbreaks (Duffy et al., 2009; Coelho et al., 2016), which could be due to male-to-female sexual transmission. It is still unknown if sexual transmission poses a different and higher risk to congenital infection than mosquito-borne transmission. Several studies have demonstrated that ZIKV replicates in the vaginal tract, and sexual transmission during the early phase of pregnancy can lead to birth defects in mice (Yockey et al., 2016; Duggal et al., 2017; Uraki et al., 2017; Winkler et al., 2017b). For instance, it was shown that vaginal infection of pregnant mice can trigger ZIKV infection of the fetus and promote brain developmental abnormalities (Yockey et al., 2016). When wild type dams were infected with ZIKV into the vagina embryos exhibited growth defects, although viral RNA could not be detected in the placenta and fetal body (Yockey et al., 2016). However, when type-I IFN knockout dams were subjected to intravaginal infection, higher levels of ZIKV RNA were observed in the placenta and fetal body (Yockey et al., 2016). Infection of type-I IFN knockout dams with ZIKV was more deleterious when it was done at E4.5, as all fetuses were reabsorbed, whereas when infection took place at E8.5, fetuses survived but developed reduced body weight (Yockey et al., 2016). Moreover, sexual transmission of ZIKV between infected male and female mice that lack type-I IFN receptors resulted in restricted growth and ocular malformations in fetuses (Uraki et al., 2017). These data highlight the importance of the innate immune response in controlling ZIKV proliferation following intravaginal infection. In addition, these results also demonstrate that intravaginal infection is a potential route for fetus infection, especially in the first trimester of pregnancy.

It is still unclear whether sexual transmission could facilitate ZIKV spread in the vagina and uterus and what factors could contribute to this infection. Notably, a recently published study indicates that sexual transmission promotes significantly greater morbidity and mortality of AG129 female mice as well as higher ZIKV titers in the female reproductive tract as compared to sub-cutaneous and intravaginal infection (Duggal et al., 2018). Moreover, the percentage of ZIKV positive fetuses was higher when females were infected sexually (88%), as compared to sub- cutaneous (50%) or intravaginal (53%) inoculation routes (Duggal et al., 2018). These data suggest that sexual transmission could account for some of the fetal alterations observed even within *Aedes* endemic regions. Further studies will be determine whether an ascending infection from the vagina to the fetus provides an easier path for fetal infection than a mosquito bite, or whether the two events combined would facilitate the development of CZS.

## Conclusion

The 2015 ZIKV outbreak caused an alarming situation linking the mosquito-borne flavivirus to devastating birth defects, such as microcephaly (unusually smaller-than normal size of the head). ZIKV seems to be the first flavivirus with the unique ability to traverse, infect and damage the placenta, which is resistant to most pathogens, as traces of the virus have been confirmed in the central nervous system and amniotic fluid of the affected babies. Although, many promising findings begin to uncover the complex role of the placenta in the pathobiology of ZIKV infection, several important events linking ZIKV infection to mammalian brain growth deficits still remain poorly characterized. Evidently, more extensive and long-term studies unraveling the major complications of fetal injury and death *in utero* are warranted. Considering the significant role in ZIKV-related fetal abnormalities, understanding placenta-induced vertical transmission and fetal brain injury would be valuable. Some of the challenges involved in studying placental infection and damage in model systems include isolation of placental primary cells/trophoblasts, morphological differences between murine and human placentas, and distinct placental inflammatory responses among mammals. In addition, most models of ZIKV infection during human pregnancy investigate the second or the third trimester placentae that differs both, in structure and cellular composition, from the first trimester placentae, which is the period mainly associated with intrauterine growth restrictions, severe microcephaly and ocular abnormalities.

Coordinated interdisciplinary studies are needed to understand the sudden emergence and asymmetric geographic distribution of CZS among ZIKV infection cases. Studies deciphering the impact of ZIKV transmission and CZS prevalence are often confounded by underestimation of ZIKV infected individuals, poor differential diagnosis between microcephaly and intrauterine growth restriction, and immunological cross-reactivity among flaviviruses. Whether infants exposed to ZIKV infection postnatally will experience any prolonged negative outcomes is still unknown, so the close long-term surveillance and treatment of potentially affected infants and their families will be necessary.

## Author Contributions

SFK wrote the sections “Introduction”, “Epidemiology”, part of “Modes of Transmission” and “Conclusion.” FMR wrote parts of the section “Modes of Transmission.” TU wrote the section “Genomic Structure.” EVM created the figures. AAR made intellectual contribution into the discussion. SCV contributed to overall supervision of the review process, editing of the manuscript, and managing several research teams.

## Conflict of Interest Statement

The authors declare that the research was conducted in the absence of any commercial or financial relationships that could be construed as a potential conflict of interest.
